# Towards Determining Biosignature Retention in Icy World Plumes

**DOI:** 10.3390/life10040040

**Published:** 2020-04-16

**Authors:** Kathryn Bywaters, Carol R. Stoker, Nelio Batista Do Nascimento, Lawrence Lemke

**Affiliations:** 1 SETI Institute, Moffett Field, CA 94043, USA; 2 NASA Ames Research Center, Space Science Division, Moffett Field, CA 94035, USA; nelio.b.nascimentojr@nasa.gov (N.B.D.N.J.); lglemke@gmail.com (L.L.)

**Keywords:** icy world, plume, life detection, microbes, lipids, Europa, Enceladus, astrobiology

## Abstract

With the discovery of the persistent jets of water being ejected to space from Enceladus, an understanding of the effect of the space environment on potential organisms and biosignatures in them is necessary for planning life detection missions. We experimentally determine the survivability of microbial cells in liquid medium when ejected into vacuum. Epifluorescence microscopy, using a lipid stain, and SEM imaging were used to interrogate the cellular integrity of *E. coli* after ejected through a pressurized nozzle into a vacuum chamber. The experimental samples showed a 94% decrease in visible intact *E. coli* cells but showed a fluorescence residue in the shape of the sublimated droplets that indicated the presence of lipids. The differences in the experimental conditions versus those expected on Enceladus should not change the analog value because the process a sample would undergo when ejected into space was representative. *E. coli* was selected for testing although other cell types could vary physiologically which would affect their response to a vacuum environment. More testing is needed to determine the dynamic range in concentration of cells expected to survive the plume environment. However, these results suggest that lipids may be directly detectable evidence of life in icy world plumes.

## 1. Introduction

In the search for life outside of Earth it is necessary to quantify the potential abundances of biosignatures, including whole single-celled organisms, in target environments. Sample collection techniques aside, which can also affect the integrity of the biosignatures being collected, the physical processes of the target environment can have an effect on the retention of biosignatures. Knowledge of the types and expected quantities of these biosignatures will enable detection strategies and sensitivity limits to be set for proposed life detection instrumentation. To this end, an understanding of the effect of the physical environment on organisms and biosignatures is essential.

One planetary environment that is of growing interest for life detection missions are icy moons, particularly Enceladus and Europa. Enceladus has a sub-ice-shell global ocean [1] of salty water [2]. It is estimated that the ocean is 26–31 km thick, beneath a 21–26 km thick icy crust [1]. The discovery, by the Cassini Saturn orbiter mission, of plumes of water carrying fine icy particles venting from four long fractures in the south polar region of Enceladus [3,4,5,6], along with evidence indicating ongoing hydrothermal activity [7], has focused attention on sampling the potentially habitable environment of the subsurface liquid water on that body [8,9,10,11]. The persistent jets of water being ejected provide a source of potentially life-bearing material that is emitted to space.

The habitability of the Enceladus subsurface ocean has been inferred from the Cassini orbiter which sampled plume-derived material. Cassini detected frozen droplets of water [4] with salinity comparable to Earth’s ocean suggesting the water is in contact with a rocky core [12]. Both H_2_ and CO_2_ have been detected in the plume, as have organic carbon molecules [13,14]. Nitrogen was also present as NH_3_ which is readily used by microbes. The major elements needed for life (C, H, N, O, P, S) have been detected or are expected to be present [15]. McKay et al. [15] conducted an extensive consideration of the habitability of the Enceladus subsurface ocean, including a breakdown of the requirements for life, limits of life and how the Enceladus ocean compares. Based on these considerations, it seems likely that the subsurface ocean of Enceladus would be habitable to terrestrial microbes if introduced there, although whether the conditions allowed for the origin of life on Enceladus is an open question [16].

The potential habitability of icy moons has led to studies addressing possible microbial survival and growth [17,18,19,20,21,22], as well as estimates of biomass concentrations [23,24,25] in these environments. The results show that, under certain conditions (degree of convection, mantle rheology, etc.), there would be enough chemical energy to sustain an ecosystem of chemoautotrophic organisms. The Europa Lander Study 2016 Report [23], using Earth analogue environments (i.e., subglacial lakes), placed the upper range of cell density at approximately 100 cells per mL and set that value as the limit of detection for life detection instruments.

Neveu et al. [26] presented NASA’s Life Detection Ladder, which outlined the methods for extant life detection beyond Earth. One of the given criteria for a convincing life-detection measurement was that the measurement context must be sufficiently “survivable”. The term survivable was used to address the issue that physical, chemical, and geological conditions in the environment that the sample encounters had not destroyed the targeted signs of life. Here we investigated the survivability of microbial cells in simulated icy world plumes as they are introduced into the highly desiccating conditions of vacuum. This work begins to experimentally deduce the survivability and thereby the potential abundances of organisms/biomarkers that could be expected in these environments.

**Figure 1:**
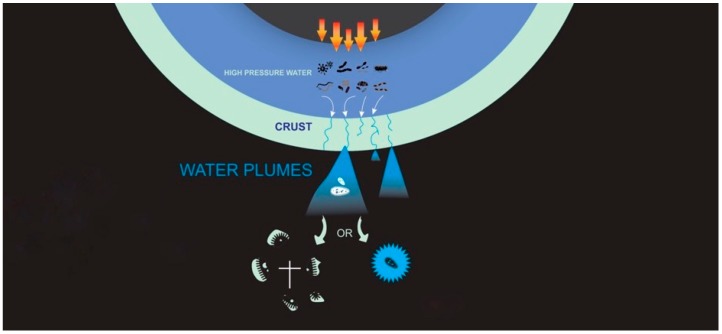
Schematic depicting hydrothermal heat transfer from an icy moon core to liquid water. Cracks in an icy crust overlying the liquid allows plumes that erupt into space potentially carrying cells and/or biomarkers.

If there is life in the oceans of icy moons that is being injected into space by the plumes (Figure 1), it becomes imperative to quantitatively understand the effects of this process on the integrity of cells and potential biosignatures. Here we investigate what would happen to a specific kind of terrestrial microbes inhabiting a liquid water environment if they were injected into vacuum. We measure the physical response of microorganisms injected by plumes into the highly desiccating conditions of vacuum. It has been established that biomolecules and even organisms can survive space vacuum [27,28] even though they experience dehydration and cellular damage [29,30]. Simple and complex organic molecules have also been shown to be ubiquitous in space [31,32]. However, the retention rate of intact cells and degree of cellular structural damage has yet to be described or quantified.

## 2. Materials and Methods

*Escherichia coli* (*E. coli*) was selected as the organism for testing. There are a wide range of model organisms from different target environments that could have been tested, such as a methanogen as this metabolism could harness the energy flux present on Enceladus [14], *Nautilia profundicola*, an anaerobic nitrate-reducing bacterium from an active seafloor vent, *Colwellia psychrerythraea*, a heterotrophic marine bacterium ubiquitous in cold marine ecosystems, or *Bacillus pumilus* a spore-forming bacterium that have shown especially high resistance to ultraviolet (UV) radiation. However, for the scope of this work we selected *E. coli* as a point of reference by which to begin measuring the effects of the plume environment on cell retention due to the vast body of work that has already been conducted on this organism.

*E. coli* strain OP50 was maintained by weekly transfers to LB Broth (LB Broth: (1) 0.17 M NaCl (Fisher; S271), (2) 5 g yeast extract (Alfa Aesar; H26769), and (3) 10 g tryptone (Fisher; BP1421) added to 0.8 L of DiH_2_O. The pH was adjusted to 7.5 with NaOH, filled to 1 L, and then autoclaved). Cells were grown to late log phase and harvested by centrifugation at 6000 rpm for 10 min. Cells were washed to remove the LB broth (due to high intrinsic fluorescence) for all experiments. The cell samples were centrifuged for 10 min in 1.5 mL tubes. Being careful not to disturb the cell pellet, the supernatant was then removed and cells were resuspended in PBS by quickly vortexing (PBS: (1) 0.14 M NaCl (Fisher; S271), (2) 0.003 M KCl (Sigma; P-9333), (3) 0.01 M Na_2_HPO_4_ (Fisher; BP332), and (4) 0.002 KH_2_PO_4_ (Sigma; P5379) added to 0.8 L of DiH_2_O. The pH was adjusted to 8.3, filled to 1 L and then autoclaved). This was repeated for a total of three washes. Growth media and PBS buffer were made using ultra high purity water. A pH range of 7.5–8.3 was used: (1) to ensure *E. coli* cells were not disrupted and (2) because it is reasonable for seawater on Earth. A wide range of pH values have been proposed for Enceladus, potentially > 10 [33] and as low as 2.6 for Europa [34]. However, this paper only provides a first quantitative data point and more experimentation is needed.

Primuline was selected as the fluorescence stain due to the fact that it is a non-destructive lipid stain. The case has been made that lipids are the universal biomarkers of extraterrestrial life [35] and therefore lipids are likely candidate measurement targets for a life detection mission. The use of a lipid stain also allowed for the visualization of the cellular membranes and helped determine if they had remained intact (this does not differentiate between live/dead cells). Primuline (MP Biomedicals 195454) was added to cells resuspended in the PBS buffer, to achieve cell concentration of ~10^5^ cells/mL, at a concentration of 100 μM. A cell concentration of ~10^5^ cells/mL was chosen to ensure that a sufficient number of cells would be captured in the sample collectors. Cell/dye combination was prepared in 50 mL Falcon tubes, quickly vortexed for mixing and then incubated for 10 min at room temperature in the dark. After the incubation period, stained cells were then washed again.

**Figure 2:**
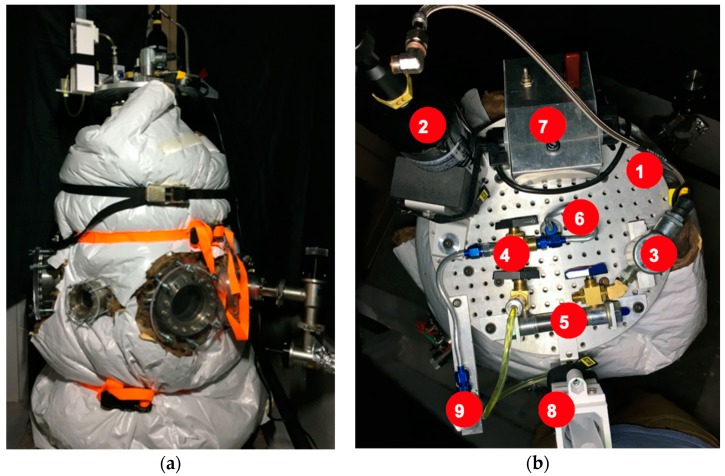
(**a**) Experiments were performed in a vacuum chamber with a 463 mm diameter (473 L), evacuated with a roughing pump. (**b**) Image of injection system. (1) Injection Deck, (2) Gas Bottle, (3) Solenoid vale, (4) Directional valve, (5) Piston, (6) Internal nozzle feed, (7) Action Trigger, (8) Solution Reservoir, and (9) External test nozzle.

**Figure 3:**
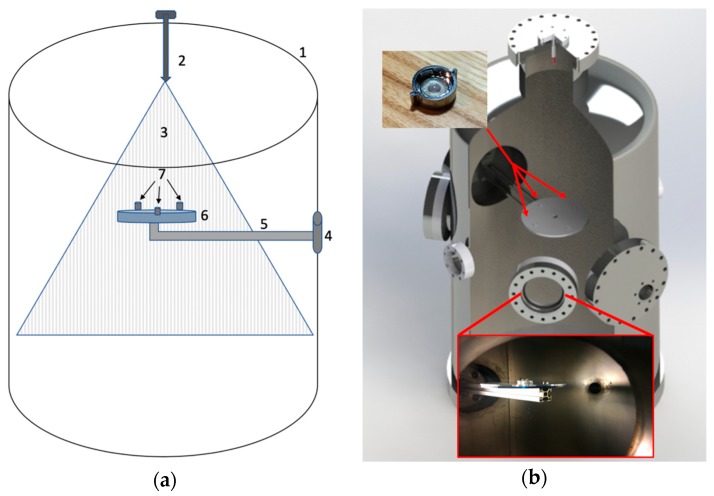
(**a**) Simplified illustration of the chamber geometry. The parts are (1) the outer volume of the chamber, (2) nozzle and tubing, (3) spray pattern, (4) flange, (5) arm, (6) plate holding sample collectors, and (7) SEM collection cups. (**b**) Sample collectors (photo insert upper left) were mounted on a custom-made acrylic plate (red arrows indicate where they were mounted) that was fixed to an arm mounted on one of the flanges. Photo insert at bottom shows the arm and plate inside the chamber.

Experiments were performed in a vacuum chamber of 463 mm diameter (473 L) (Figure 2). Experiments were performed at starting pressures between 8.00 and 8.67 Pa. The chamber was depressurized using a Sargent Welsh Direct Torr 8851 roughing vacuum pump. Sample collectors (SEM capsules and microscope slides) were mounted on a custom-made acrylic plate that was fixed to one of the flanges in the chamber (Figure 3).

Injection of *E. coli* was performed using a high-pressure nozzle system. A double-acting pneumatic piston/cylinder (Bimba, PN 0074-DXP) was actuated via a short pulse of high-pressure CO_2_ gas supplied at 5.8 MPa to force *E. coli* containing fluid samples through an injection nozzle. High injection pressures were needed to ensure the smallest mean droplet size which was 35 μm for the ceramic nozzle per the manufacture’s specification. Two different hollow-cone type nozzles were experimented with: a ceramic misting nozzle (Aeromist Inc. Phoenix Arizona ¼” NPT Ceramic Nozzle SKU: 52252 rated ≈ 5.1 L/h) or a stainless-steel nozzle (Aeromist high pressure anti drip 0.012X 10/24 SKU: 51433 rated ≈ 6.0 L/h). Both nozzles had orifices of 0.3175 mm in diameter. The nozzle in use was located on axis at the apex of the vacuum chamber, pointing downward toward the collector plate. The piston/cylinder assembly was configured to act as a metering pump. To charge the cylinder, a ball valve was manually opened, and 5 mL of test fluid was manually injected into the base port of the cylinder from a sterile syringe, causing the piston to displace to its limit in one direction. During injection into the vacuum chamber, the ball valve was closed and CO_2_ pressure was directed through the nose cylinder port to the opposing face of the piston by an electrically operated on/off solenoid valve (Peter Paul Electronics model 19052), causing the piston to displace to its limit in the other direction, thereby forcing the metered fluid through the nozzle.

Pressure in the chamber was monitored with a convection gauge sensor tube (ConvecTech Part CVT-275-101) and controller (Terranova 906A) convection gauge. A humidity sensor (Adafruit BME280 Temperature Humidity Pressure sensor) was mounted under the sample holder.

Prior to injection of fluids containing bacteria, the internal chamber was cleaned with ethanol and the entire steel chamber was heated to sterilize by wrapping it with heat tape and covering with a Rockwool insulating blanket. The chamber temperature was measured at 5 different locations with an average temperature of 149 °C and maintained at this temperature for 12 h. The sample holders were cleaned using ethanol directly before insertion into the chamber.

For sample collection, QuantomiX WETSEM^®^ (QX-102) capsules were selected for use because they do not require any treatment prior to viewing. 3 WETSEM^®^ capsules were mounted along with 3 microscope slides on the sample collection plate (Figure 2). SEM capsules and microscope slides were mounted so that typical areas of the chamber were sampled to account for potential variability in sample distribution. Injections of fluid into the chamber consisted of: (1) a negative control; no *E. coli* (under vacuum), (2) a positive control; *E. coli* under standard atmospheric pressure and (3) the experimental sample; *E. coli* under vacuum. The collection area of the SEM capsules was 28.27 mm^2^ and 645.16 mm^2^ for the microscope slides.

**Figure 4:**
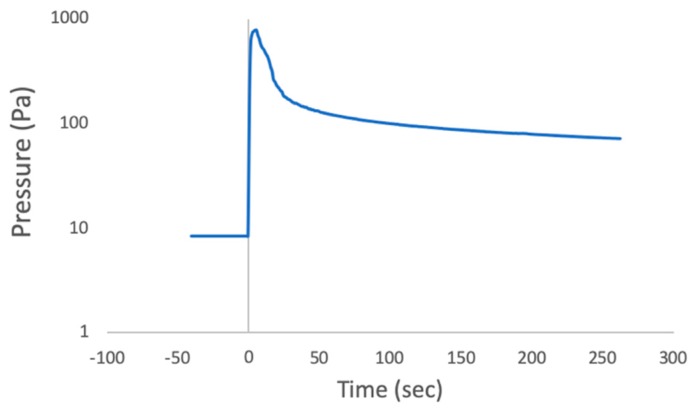
Pressure (Pa) in the chamber before and after injection; time of injection T = 0. Primary data is available in Excel file S1.

Chamber pressure prior to injection was between 8.00–8.67 Pa. This pressure was the practical minimum achievable with the available apparatus but was found to be sufficient to produce rapid or flash freezing of the injected drops. Upon fluid injections the injected material froze rapidly, then the pressure rose in the chamber because evaporation of the water drops produced water vapor that quickly filled the small chamber volume to a typical value of ~800 Pa (Figure 4). After fluid was injected into the chamber, the vacuum pump was restarted, and pumping continued until drops had fully sublimated whence the samples were removed from the chamber. For comparison with the vacuum sublimated ice drops, the water drops from positive controls, after being removed from the chamber, were allowed to dry before imaging.

There were experimental differences that affect the fidelity of this experiment to ocean world plumes. Table 1 compares the experimental parameters for these to those measured or expected at Enceladus.

Epifluorescence microscopy was conducted on the microscope slides and imaged using a Leica DMi8 S Platform with a 63X objective (Leica HCX PL APO) and a filter set with 327–402 nm excitation passband, 409 DM, and 417–477 nm emission passband. The WETSEM^®^ capsules were imaged using a Hitachi S-4800 tabletop SEM, resolution 2 nm at 1 kV. Three samples were collected on microscope slides for each of the negative control, positive control and experimental sample. Cell counts were performed via epifluorescence microscopy on each microscope slide per sample, counting areas were 0.15 mm^2^. Using ImageJ each cell was selected, and area traced by hand to delineate between nearly intact and damaged/destroyed cells. Any selected cell with a surface area of less than 0.3 μm^2^ was not counted.

## 3. Results

There was a difference in the performance between the two different nozzle types used. Cell counts of positive controls (*E. coli* under standard atmospheric pressure) injected into the chamber using the ceramic vaporizer nozzle and stainless-steel nozzle were compared and showed a significant decrease in the cell retention with the stainless-steel nozzle. Only approximately 2% of all visible intact *E. coli* cells were preserved using the stainless-steel nozzle (Figure S1). All experimental parameters were consistent between the use of the ceramic vaporizer nozzle and stainless-steel nozzle with the only variable being the nozzle type. The ceramic nozzle showed no degradation in *E. coli* cell counts by comparison. Therefore, the experiments used only the ceramic nozzle and those results are reported here.

Pressure in the chamber was monitored and recorded (Figure 4). At the start the chamber contained room air. The vacuum pump took ~2 h to achieve our target value (8.67 Pa). When the sample was injected pressure reached 797.27 Pa before decreasing again. During and immediately after injection into the chamber, it was repeatedly witnessed that only under the experimental conditions the droplets which were visible with the naked eye rapidly froze: small drops bounced off the collection plate and larger drops quickly froze on it.

**Figure 5:**
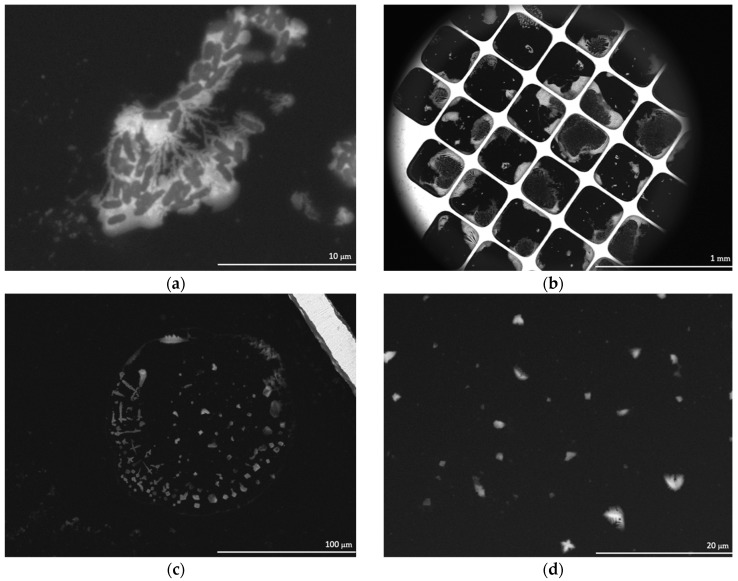
SEM images of (**a**) a positive control; *E. coli* under atmospheric pressure, (**b**) an overview of the WETSEM^®^ viewing window for the experimental samples; *E. coli* after being injected into vacuum, (**c**) the residue from a droplet in the experimental sample, and (**d**) a close up of the residue from a droplet in the experimental sample.

The SEM images of positive controls (under atmospheric pressure) using the ceramic nozzle showed *E. coli* cells intact (Figure 5a). The extent of the damage, if any, to the integrity of the cell walls could not be determined, however, the overall appearance of the cells was that they were intact. Contrary to the results seen in the positive controls, the SEM images of *E. coli* injected into the chamber under vacuum (8.67 Pa) showed a substantial decrease in visible intact cells. However, a residue appeared which outlined the droplets (Figure 5b–d). The water in the droplets had sublimated (due to being under vacuum), leaving behind what was believed to be cellular remnants—The residue observed in the SEM images.

**Figure 6:**
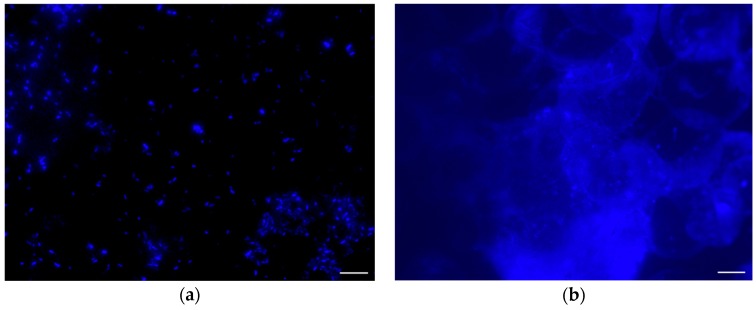
Fluorescence images of *E. coli* stained with Primuline; (**a**) a positive control; *E. coli* under atmospheric pressure; (**b**) the experimental samples; *E. coli* after being injected into vacuum. Scale bar = 10 μm. The other images for the positive control and experimental samples are available in Figure S2.

**Figure 7:**
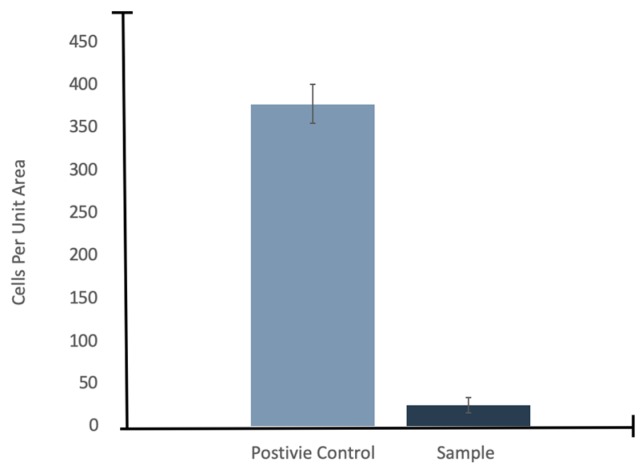
Cell counts for *E. coli* under atmospheric pressure (positive control) and under the experimental conditions, *n* = 3.

To confirm if the residue in the SEM images was in fact cellular biomarkers, *E. coli* was stained with Primuline prior to injection into the chamber and then imaged with an epifluorescence microscope. The fluorescence images of *E. coli* stained with Primuline were consistent with the SEM images (Figure 6). The positive controls showed *E. coli* cells intact with little to no stain fluorescence outside of the cell structures (little background fluorescence). The experimental samples showed a fluorescence residue in the form of the sublimated droplets indicating the presence of lipids, with only few intact cells observed. Cell counts performed showed that there was a 94% reduction, with an uncertainty of 6%, in visible intact *E. coli* cells when subjected to injection into the vacuum chamber under experimental conditions (Figure 7).

## 4. Discussion

Any putative life possible in the interior ocean of an icy moon is adapted to a liquid water environment. It is important to understand how the process of being ejected into the vacuum of space would impact such life in order to design missions and choose the best instruments for detecting it. Our work has focused on *E. Coli*, a specific aqueous organism, because it has been well studied. This work has allowed us to gain insight into the impact on cell membranes of being rapidly frozen. Much more extensive experiments could and should be done to explore alternative types of organisms such as methanogens, and to investigate a wider range of environmental conditions such organisms may experience in their native habitat. Still, this preliminary work helps to bridge the current knowledge gap between the modeled abundances of cells in the liquid water environment of icy world plumes and experimentally derived values of what to expect at collection.

It is important to understand how the experimental conditions differ from those of the Enceladus plumes to ensure that the results are relevant. The chamber pressure prior to injection (8.67 Pa) was not chosen to match conditions in the Europa plume but rather was the lowest pressure that we could achieve using the available apparatus. Additionally, after injection the water drops immediately began to evaporate into the small volume chamber causing the pressure in the chamber to increase to 799.94 Pa of water vapor. The liquid water evaporated when injected because the pressure in the chamber was below the triple point pressure of water, so water boiled until its temperature reached 0 °C. The evaporation is a phase change that requires heat (the latent heat of vaporization) which was pulled from the drops quickly, bringing their temperature to the freezing point. Removing the water vapor from the chamber using the vacuum pump took considerable time (Figure 4). In the case of Enceladus plumes, the water vapor evaporating from plume drops would be expanding into the unlimited volume of space. While there would be a short period during eruption when the drops would experience higher gas pressure from the water vapor, it would drop very quickly to essentially zero. Compared to an icy world plume of water drops, the high-water vapor pressure in the chamber after injection may have slowed the rate of evaporation of the drops, and therefore slowed the rate of freezing. Nevertheless, large drops were observed to freeze within 2 s and to remain frozen after the pressure increased and until the experiment was stopped. Our apparatus was not cooled so the internal components such as the sample collectors were at room temperature when the fluid injection occurred. Since these components had much greater thermal mass than the ice particles, the ice was continuously heated by the sample capture components causing them to sublime fairly quickly, in effect freeze drying the material in the ice particles. So, in the final state before the chamber was opened, this material, including cells, was both in vacuum and dry. In the Enceladus plume, the ice particles once ejected equilibrate to their equilibrium temperature at the orbit of Saturn and so sublimate very slowly.

While the environmental conditions produced in the lab experiment are different in detail than what occurs when water drops erupt from Enceladus, our results are still a valuable analog providing insight into the fate of any microbes carried by the plume drops. The process that water drops undergo when ejected into space was simulated as the drops quickly froze due to the heat loss during vaporization. This rapid freezing is likely responsible for the destruction of cells causing cell lysis. Rapid freezing will certainly occur if liquid water is erupted into vacuum such as occurs in the Enceladus plume. However, this difference should not change the analog value of the experiment because the process a sample would undergo when ejected into space was largely duplicated with flash freezing followed by slow sublimation.

From the results presented, it is possible to estimate the potential cell concentrations that might be observed in icy world plume ejecta. There are now several lines of evidence suggesting the existence of plumes at Europa’s surface [38,39,40]; taking this into account and considering the upper range of cell density of approximately 100 cells per mL derived in the Europa Lander Study 2016 Report [23] combined with our result of a 94% reduction in visible intact *E. coli* cells, this would mean that the amount of detectable intact cells would be 6 cells per mL. However, the assumption here is that cells on Europa would respond in a similar way as *E. coli*, which may not be the case. For Earth organisms, we know that there is a wide range in composition and lysability of cell walls and membranes which would affect their response to a vacuum environment. More testing, with a multitude of different organisms, is needed to determine the dynamic range in concentration of cells expected to survive the plume environment.

Intact cells might have a low retention rate in plume ejecta; however, our results show that ejection of *E. coli* from a liquid medium into vacuum causes cell lysis, leaving behind a residue of lipids and potentially other biomarkers such as proteinogenic amino acids. From the lipid residue observed via epifluorescence microscopy, it is reasonable to assume the lipid structure itself is still intact along with other cellular non-polar molecules, at least for a large portion of the sample, even if the cell membrane is not. Presuming intact lipids, the needed concentration to meet the values proposed for the lower limit of detection in the Europa Lander Study 2016 Report [23] would still be a viable option as a biomarker for a life detection mission. This result suggests that directly detectable evidence of life in plumes erupted from the ocean of icy moons may be lipids.

However, further characterization of the remaining material after exposure to vacuum is warranted to understand if the phospholipid molecules are still intact and, if not, are hydrocarbon tails of these lipids (i.e., fatty acids/isoprenoids) distinguishable from abiogenic sources of these molecules. High resolution microscopy and gas chromatography-mass spectrometry (GC-MS) analysis would enable the sample to be interrogated for cellular damage and identification of lipid molecules, respectively. Three key technologies needed for the unambiguous detection of life have been identified; organic analyzer (GC-MS), vibrational spectrometer, and a microscope [23]. Employing these technologies for characterization of the sample could potentially aid in the design of future missions.

Our initial testing discovered that cells were destroyed in passage through the stainless-steel nozzle but not through the ceramic nozzle. The stainless-steel nozzle was originally selected because it yielded a mean droplet size (≈10 micrometer) near the target size range of Enceladus’ particles, when operated at its rated pressure. Destruction of cells in the stainless-steel nozzle was unexpected and showed that the positive control experiments were needed to characterize the behavior of the system. Both nozzle assemblies have spring loaded balls sitting in shaped valve seats that serve as check valves to allow flow in the forward direction only. When no line pressure is applied to the inlet, the seated ball also prevents drips. When sufficient line pressure is applied, the pressure unseats the ball and allows fluid to flow to the discharge orifice. The nominal unseat pressure for the stainless-steel nozzle is ≈ 1380 kPa, while the unseat pressure for the ceramic nozzle is ≈ 240 kPa. This implies that the clearance between the ball and the valve seat is smaller on the stainless valve than on the ceramic valve when fluid is flowing and may have forced the cells through an opening smaller than the cell diameter or generated an internal pressure outside the range *E. coli* could tolerate. We note this for completeness in case our experimental method is replicated by others. Because the ceramic nozzle did not destroy the cells in passage through the orifice, the ceramic nozzle was used in all data gathering runs even though it produced a somewhat larger mean droplet size (≈35 micrometer).

The Enceladus plumes have particles sizes in the range 1–10 microns [37], the lower end of this range being comparable to the size of an individual microbe. A conceptual study by Porco et al. [37] discusses processes involving the formation and rising of bubbles in the Enceladus ocean thought to create the plume particles. The rising of bubbles to the surface creates drops of different sizes. These bubbles are expected to concentrate the organics and microbial numbers compared to the bulk fluid, with the largest drops having the highest concentrations. These largest are called jet drops; they form as a rebound process after gas filled bubbles burst and they can be as large or larger than the drops in our experiments. The process that we used to produce drops (misting nozzle) is fundamentally different than the process producing them in the plumes. However, this should not materially impact the rapid freezing process that we believe causes cell lysis. The size distribution observed in the Enceladus plumes also does not represent the range of sizes produced by the bursting bubbles, as the largest particles would fall rapidly back to the surface, and the remaining ice particles would decrease in size as they sublimate.

## 5. Conclusions

This work has started to fill in the uncertainties about the survivability of biosignatures in icy world plumes. Flash freezing of the cells, caused by the cooling effect of sublimation in vacuum caused lysing of the cell and thereby the destruction of the cell as a whole. The 94% reduction in visible intact *E. coli* cells suggested that conditions in the environment that the sample encounters has destroyed the cells to some degree. However, to fully understand how ejection of cells living in a liquid environment into vacuum impacts the potential samples for life detection analyses, it would be necessary to investigate the response of multiple different types of microorganisms to the physical processes undergone in the plume environment. Even in the absence of intact cells, other viable biosignatures could still persist in the plume ejecta, particularly in the form of lipids.

## Figures and Tables

**Table 1:** Comparisons of experimental parameters to those measured or expected at Enceladus.

Parameter	Measured or Expected at Enceladus	Experimental
Pressure	1.3 × 10^−9^ Pa [33]	8.67 Pa
Temperature	273 K [36]	296 K
pH	9–11 [33]	7.5–8.3
Injection velocity	100 m/s	20 m/s
Droplet size	1–10 μm [37]	35 μm
Cells	100 cells/mL (for Europa)	~10^5^ cells/mL
Organism	Methanogen *	*Escherichia coli*

* Due to energy availability.

## Supplementary Materials

The following are available online at https://www.mdpi.com/2075-1729/10/4/40/s1, Figure S1. Fluorescence images of *E. coli* stained with Primuline. Figure S2. Cell counts for E. coli under atmospheric pressure (positive control) when the ceramic nozzle was used versus the stainless-steel nozzle, n = 3. Excel file S1: Pressure versus time for experimental trial.
