# Supplementary material for: Towards Determining Biosignature Retention in Icy World Plumes
Source: Life (Basel). 2020 Apr 16;10(4):40. doi: 10.3390/life10040040 (PMC7235855; doi:10.3390/life10040040)
Supplement: Supplementary file 1 [file life-10-00040-s001.zip › life-765288 supplementary XML/Figure S1_S2.docx]

Supplementary Figures


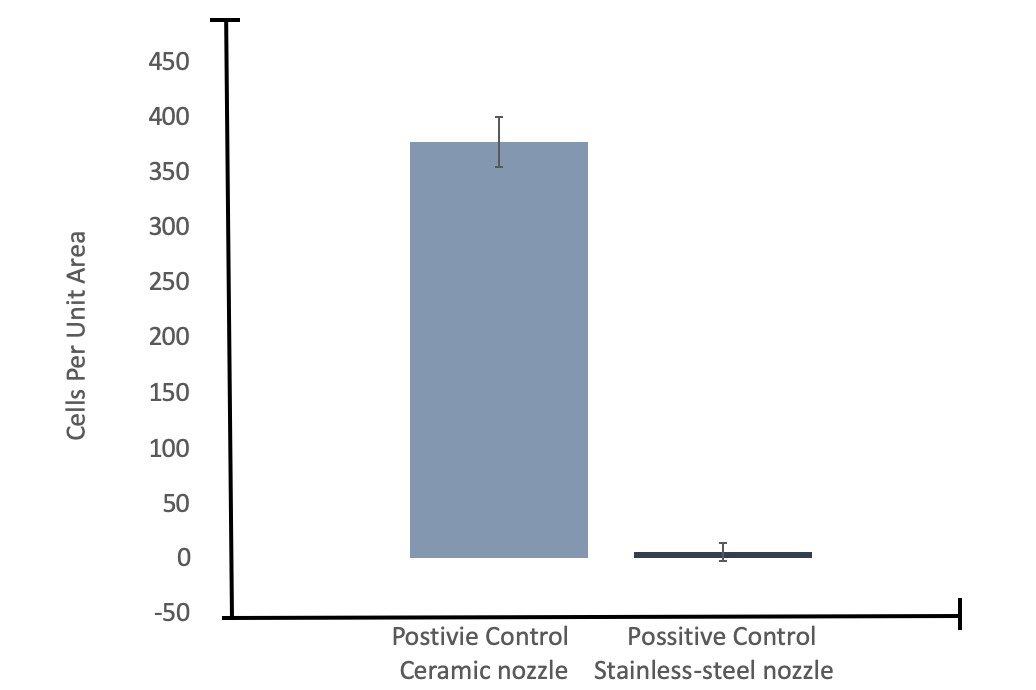


**Figure S1.** Cell counts for *E. coli* under atmospheric pressure (positive control) when the ceramic nozzle was used versus the stainless-steel nozzle, n = 3.

| 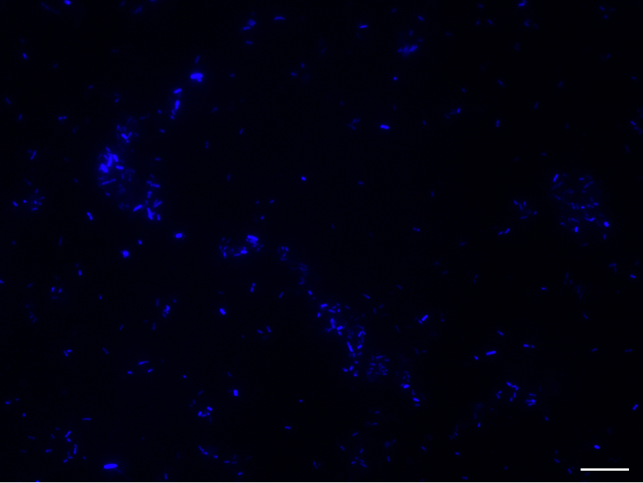  (**a**) | 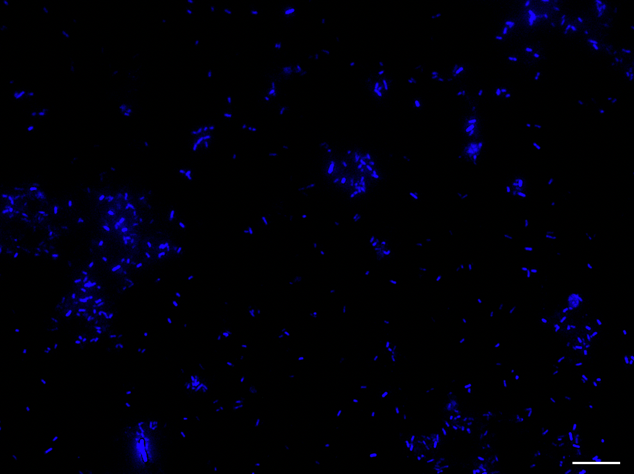  (**b**) |
| --- | --- |
| 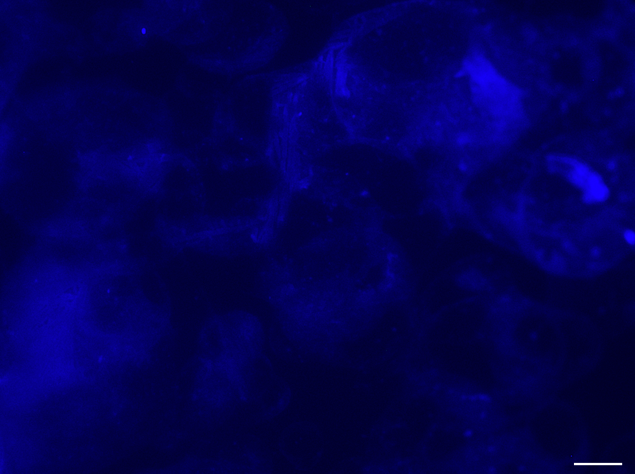  (**c**) | 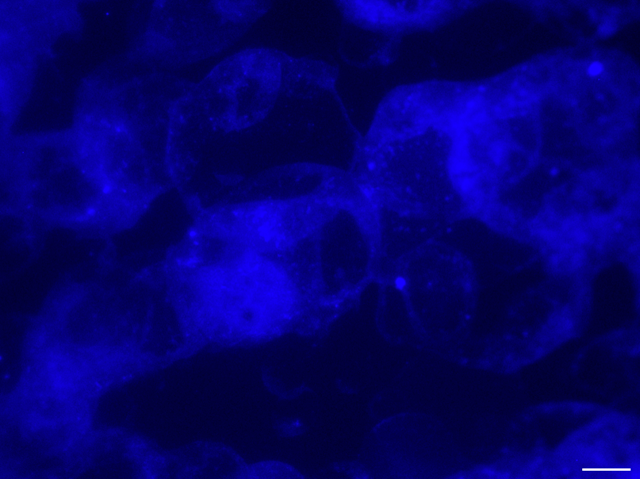  (**d**) |

**Figure S2.** Fluorescence images of *E. coli* stained with Primuline; (**a-b**) a positive control; *E. coli* under atmospheric pressure; (**c-d**) the experimental samples; *E. coli* after being injected into vacuum (65 mTorr). Scale bar = 10 um.
